# Sex-specific transcriptome differences in a middle-aged frailty cohort

**DOI:** 10.1186/s12877-022-03326-7

**Published:** 2022-08-09

**Authors:** Natasha L. Pacheco, Nicole Noren Hooten, Yongqing Zhang, Calais S. Prince, Nicolle A. Mode, Ngozi Ezike, Kevin G. Becker, Alan B. Zonderman, Michele K. Evans

**Affiliations:** 1 Laboratory of Epidemiology and Population Sciences, National Institute On Aging, National Institutes of Health, Baltimore, MD USA; 2 Laboratory of Genetics and Genomics, National Institute On Aging, National Institutes of Health, Baltimore, MD USA

**Keywords:** Midlife, Gene expression, Inflammation, Musculoskeletal, Aging

## Abstract

**Background:**

Frailty is a clinical syndrome described as reduced physiological reserve and increased vulnerability. Typically examined in older adults, recent work shows frailty occurs in middle-aged individuals and is associated with increased mortality. Previous investigation of global transcriptome changes in a middle-aged cohort from the Healthy Aging in Neighborhoods of Diversity across the Life Span (HANDLS) study demonstrated inflammatory genes and pathways were significantly altered by frailty status and race. Transcriptome differences in frailty by sex remain unclear. We sought to discover novel genes and pathways associated with sex and frailty in a diverse middle-aged cohort using RNA-Sequencing.

**Methods:**

Differential gene expression and pathway analyses were performed in peripheral blood mononuclear cells for 1) frail females (FRAF, *n* = 4) vs non-frail females (NORF, *n* = 4), 2) frail males (FRAM, *n* = 4) vs non-frail males (NORM, *n* = 4), 3) FRAM vs FRAF, and 4) NORM vs NORF. We evaluated exclusive significant genes and pathways, as well as overlaps, between the comparison groups.

**Results:**

Over 80% of the significant genes exclusive to FRAF vs NORF, FRAM vs NORM, and FRAM vs FRAF, respectively, were novel and associated with various biological functions. Pathways exclusive to FRAF vs NORF were associated with reduced inflammation, while FRAM vs NORM exclusive pathways were related to aberrant musculoskeletal physiology. Pathways exclusive to FRAM vs FRAF were associated with reduced cell cycle regulation and activated catabolism and Coronavirus pathogenesis.

**Conclusions:**

Our results indicate sex-specific transcriptional changes occur in middle-aged frailty, enhancing knowledge on frailty progression and potential therapeutic targets to prevent frailty.

**Supplementary Information:**

The online version contains supplementary material available at 10.1186/s12877-022-03326-7.

## Background

Frailty is a syndrome described as reduced physiological reserve and increased vulnerability to stressors [1]. Individuals with frailty have reduced strength and endurance, and increased risk of falls, institutionalization, hospitalization, disability, and premature mortality [1]. Frailty prevalence in adults ≥ 65 years old in the United States is estimated at 15% [2]. Health disparities have been observed in frailty; individuals from lower socioeconomic groups, racial and ethnic minority groups, and women have significantly higher frailty prevalence [2].

Research in frailty has predominantly studied older adult populations (≥ 65 years old). The few studies that have evaluated frailty prevalence in middle-aged cohorts (35–64 years old) found frailty prevalence ranges from 2–8.5% [3–6]. One of these studies found mortality was associated with frailty for all age groups (37–73 years old) except for women aged 37–45 years old [4]. We also recently evaluated frailty prevalence in the Healthy Aging in Neighborhoods of Diversity across the Life Span (HANDLS) cohort for middle-aged participants 35–64 years old and found 11% were frail [7]. Additionally, these middle-aged frail individuals had significantly reduced survival probability compared to their non-frail counterparts [7], suggesting frailty in middle age is associated with mortality. Therefore, these studies indicate frailty can occur in middle age and highlight the importance of investigating mechanisms driving frailty in middle-aged individuals.

Few studies have examined molecular mechanisms contributing to frailty pathophysiology in midlife. Using a Mendelian randomization approach, one study found individuals with reductions in low-density lipoprotein cholesterol had reduced frailty in midlife and older age [8]. Another study in African American (AA) adults aged 49–65 years old found serum proganulin levels were positively correlated with frailty and associated with a higher frailty score 9 years later [9]. We previously identified differentially expressed genes in a middle-aged frail cohort (45–49 years old) by overall frailty status and by race [10]. We discovered racial differences in gene expression were related to aberrant immune and inflammatory processes [10].

Few have examined molecular differences between middle-aged men and women, in which substantial evidence has demonstrated sex differences in the burden of frailty and frailty-associated mortality [1, 2, 11]. Women have greater frailty burden but have greater longevity compared to men [11]. Various biological factors have been suggested to contribute to sex differences in frailty, including inflammation, hormones, and genetics [11]. For example, estrogen has been linked to higher risk of autoimmunity in women, while testosterone has been implicated in decreasing immunological robustness in men [11]. It has also been suggested that women have greater physiological reserve than men [11]. How these processes are dysregulated in middle-aged frail men and women remain unknown.

Examining sex-specific molecular profiles in a middle-aged frail cohort could reveal novel insight into the sex-frailty paradox. Thus, we extended our previous study [10] and investigated transcriptome-wide changes in a middle-aged frail cohort by sex. RNA-Sequencing was utilized to identify global gene expression changes in middle-aged frail and non-frail men and women. Our pathway analyses revealed sex-specific dysregulation of key frailty-associated biological processes such as inflammation, musculoskeletal physiology, cell cycle, and metabolism.

## Methods

### Cohort description

Participants are part of the HANDLS study which has been described elsewhere [12]. HANDLS is an epidemiologic, longitudinal study examining how age-related health disparities are influenced by race, socioeconomic status, and other behavioral, psychosocial, and environmental conditions [12]. The HANDLS cohort is comprised of community-dwelling, non-institutionalized African American (AAs) and White adults between the ages of 30–64 at enrollment (2004–2009) who resided in Baltimore, Maryland [12].

A subcohort was selected for RNA-Sequencing as previously described in [10]. Briefly, 16 HANDLS participants (8 non-frail, 8 frail) were selected for RNA-Sequencing, stratified by frailty status, race (50% white, 50% AAs), and sex [10] (Table 1). Ages of the 16 participants ranged from 45–49 years old [10] (Table 1). The International Academy on Nutrition and Aging FRAIL scale (fatigue, resistance, ambulation, illnesses, and loss of weight) [13] was used to classify frail individuals, with modifications for the loss of weight domain, as previously described [7, 10]. Loss of weight was measured by responses to the following question from item two of the Center for Epidemiologic Studies Depression scale: “Over the past week did you not feel like eating or have a poor appetite?” [14]. Weight loss was categorized as “present” if participants responded “occasionally (3–4 days a week)” or “mostly (5–7 days a week)” [7]. Frailty scores were based on a composite score ranging from 0–5, where “0” represents non-frail, “1–2” represents pre-frail, and “3–5” represents frail status [7].

**Table 1:** RNA-Sequencing participant demographics

	**Women**	**Men**
N	8	8
Frail or pre-frail, N (%)	4 (50%)	4 (50%)
Age (mean ± SD)	47.85 ± 1.62	48.09 ± 1.50
African American, N (%)	4 (50%)	4 (50%)

*SD* standard deviation

### Next generation sequencing and bioinformatic analyses

Total RNA was isolated from peripheral blood mononuclear cells and library preparation, sequencing, and quality control were performed as previously described [10]. The bioinformatics pipeline is briefly outlined in Additional file 1. Raw FASTQ reads were trimmed using Trimmomatic version 0.39 to remove sequencing adapters and low-quality bases [15]. FastQC version 0.11.9 was used to evaluate additional quality control metrics for the trimmed FASTQ reads [16]. The trimmed FASTQ reads were aligned to the Ensembl human reference genome version 84 (GRCh38.p5) using HISAT2 version 2.2.1.0 [17]. The “–rna-strandness” option in HISAT2 was set to reverse strand. The resulting HISAT2 aligned SAM files were converted to sorted BAM files using samtools version 1.11 [18]. The sorted, aligned BAM files were used to create a gene counts table using featureCounts from the subread module version 2.0.1 [19]. Briefly, the Ensembl human gene annotation version 84 was used as the reference gene annotation, and the strand-specific option was set to reverse strand. Gene counts for each respective comparison group (see below) were subsetted from the original gene counts table into individual sub-tables for downstream analyses. DESeq2 version 1.30.0 was used to calculate differential gene expression [20]. Genes with a row sum count less than 10 were removed from each gene counts table prior to analyses. Pairwise comparisons were made between the following groups: 1) frail females (FRAF, *n* = 4) vs non-frail females (NORF, *n* = 4), 2) frail males (FRAM, *n* = 4) vs non-frail males (NORM, *n* = 4), 3) FRAM (*n* = 4) vs FRAF (*n* = 4), and 4) NORM (*n* = 4) vs NORF (*n* = 4) as a control. Significant genes were defined as having a fold change absolute value of ≥ 1.5, and a false discovery rate (FDR) adjusted *p*-value < 0.05.

Parametric Analysis of Gene Set Enrichment (PAGE) [21] and Ingenuity Pathway Analysis (IPA) were used to identify significant gene ontology (GO) terms. Standard Z-scores calculated using the regularized Log_2_-transformed normalized counts for all detected genes were used as input for the pathway analysis, as well as for data quality control assessment and visualizations including heatmaps. PAGE was used to identify significant gene ontology (GO) terms as previously described ([10], and references within). Briefly, PAGE GO term Z-scores were calculated based on predicting how gene expression changes in a gene set could affect a given pathway(s). A significant GO term was defined as having a minimum of 3 genes and maximum 300 genes in the gene set, and a p-value and its corrected FDR both < 0.05. IPA was utilized to identify genes enriched in canonical pathways and specific disease related function gene–gene interaction networks using the same cutoff as the significant gene selection described above. Significant canonical pathways were defined as having a -Log_10_ p-value > 1.301 (or *p* < 0.05).

## Results

### Differential gene expression with frailty and sex

**Fig. 1:**
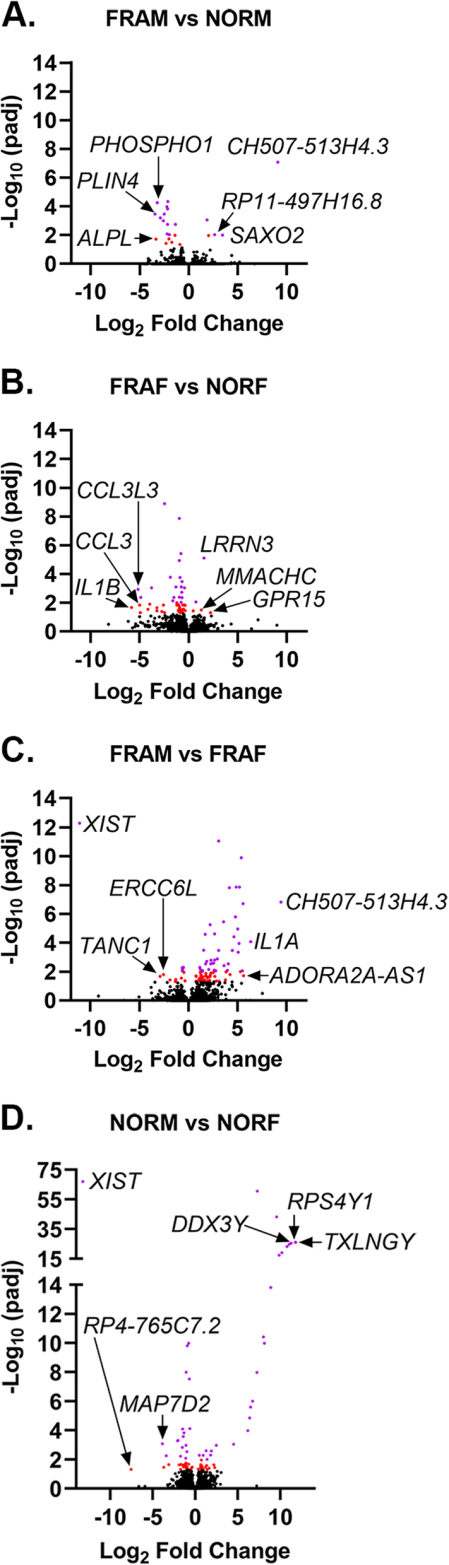
Gene expression differences associated with sex and frailty status. Detected genes from the **A**. FRAM vs NORM comparison, **B**. FRAF vs NORF comparison, **C**. FRAM vs FRAF, and **D**. NORM vs NORF comparison are plotted by Log_2_ fold change (x-axis) and –Log_10_ transformed p-adjusted values (padj, y-axis). Data points in red indicate genes significant by a padj < 0.05, and purple indicates padj < 0.01. For each comparison, the top 3 most significantly increased and decreased genes are denoted. For a complete list of all significant genes, refer to Additional files 2,3, 4, and 5

The number of significant, differentially expressed genes (DEGs) varied by frailty status and sex (Fig. 1). Twenty-three genes were significantly, differentially expressed in FRAM compared to NORM, while 47 genes were significant in FRAF relative to NORF (adjusted *p* < 0.05) (Fig. 1A-B, Additional files 2–3). The largest number of significant DEGs were found in the FRAM vs FRAF comparison, with a total of 86 DEGs (adjusted *p* < 0.05) (Fig. 1C, Additional file 4), while 78 genes were significantly, differentially expressed in the NORM vs NORF comparison (Fig. 1D, Additional file 5). Many of the significant DEGs in the FRAM vs NORM comparison group were significantly decreased in FRAM compared to NORM (Fig. 1A, Additional file 2). Similarly, significant DEGs in the FRAF vs NORF comparison were largely decreased in FRAF relative to NORF (Fig. 1B, Additional file 3). The significant DEGs from the FRAM vs FRAF comparison group had mostly increased gene expression in FRAM relative to FRAF (Fig. 1C, Additional file 4). Conversely, the ratio of significantly increased to decreased DEGs from the NORM vs NORF comparison group were similar (Fig. 1D, Additional file 5).

**Fig. 2:**
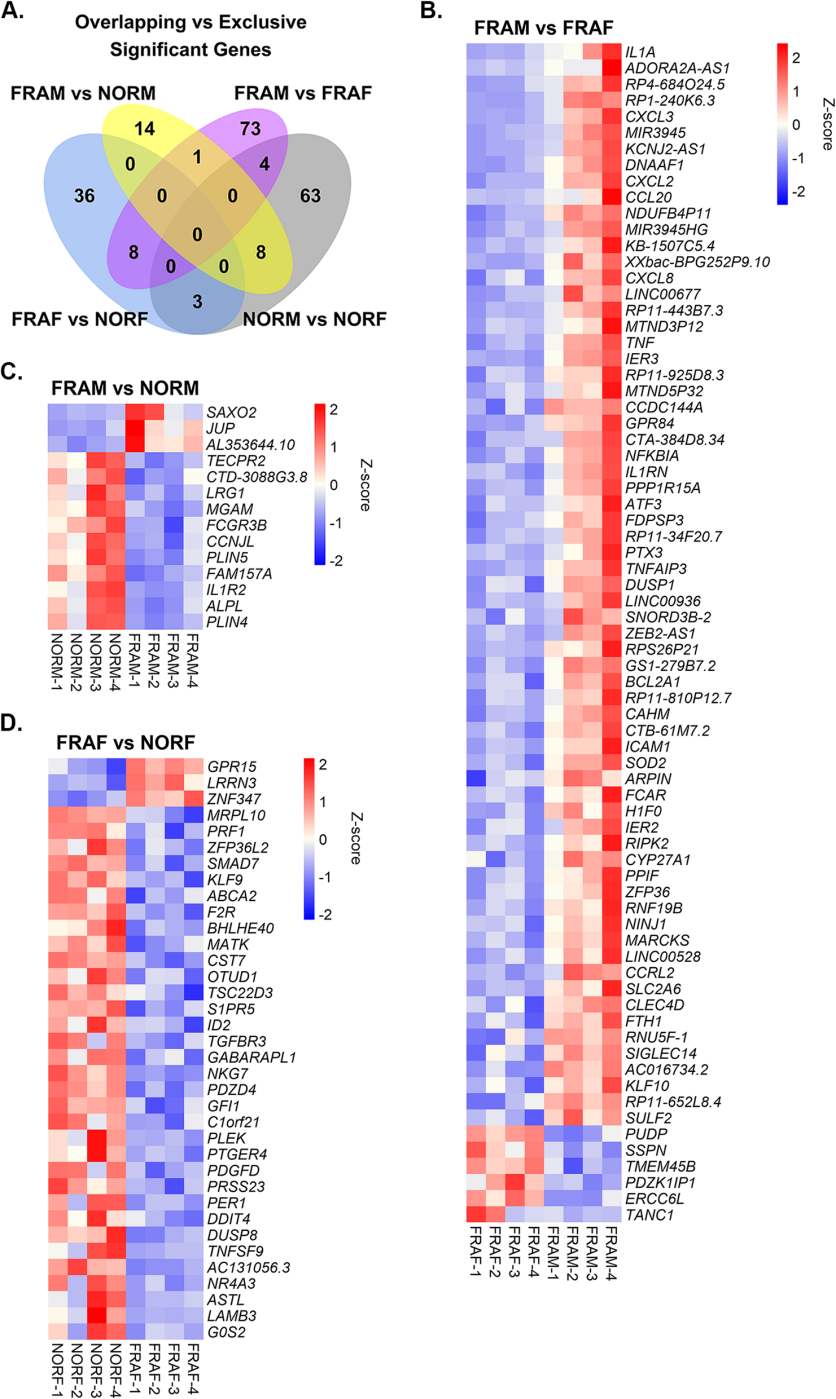
Significant differentially expressed genes exclusively identified per frailty group comparison. **A**. Venn diagram comparing lists of significant genes (Log_2_ fold change ≥ 0.58 or ≤ -0.58) identified from FRAM vs NORM (yellow), FRAF vs NORF (blue), FRAM vs FRAF (purple), and NORM vs NORF (gray) as a reference. The significant, differentially expressed genes exclusively identified in the **B**. FRAM vs FRAF, **C**. FRAM vs NORM, and **D**. FRAF vs NORF comparisons are presented as heatmaps. For each respective heatmap, columns represent individual samples and rows represent gene Z-scores. Only significant genes with a Log_2_ fold change ≥ 0.58 or ≤ -0.58 are plotted. Refer to Additional files 2, 3, 4, and 5 for complete list of significant genes

To identify significant DEGs that could potentially distinguish sex-specific differences in frailty pathophysiology, we compared the lists of significant DEGs from each respective comparison group (including NORM vs NORF as a control comparison group) for overlapping and exclusive DEGs among the 4 comparison groups (Fig. 2A). No significant DEGs were shared among all 4 groups, nor were any DEGs shared between FRAM vs NORM, FRAF vs NORF, and FRAM vs FRAF comparison groups (Fig. 2A). Additionally, there were no overlapping DEGs between the FRAM vs NORM and FRAF vs NORF groups. Only 1 DEG, *CH507-513H4.3*, was shared between FRAM vs NORM and FRAM vs FRAF (Fig. 2A). *CH507-513H4.3* is a long noncoding (lnc) RNA and was up-regulated in FRAM relative to NORM (9.1 Log_2_ fold change) as well as in FRAM compared to FRAF (9.4 Log_2_ fold change) (Additional file 2, Additional file 4). Eight DEGs were shared between the FRAF vs NORF and FRAM vs FRAF comparison groups (Fig. 2A). Six out of the 8 shared DEGs were associated with inflammation (*HLA-DPA1*, *IL1B*) and chemokine signaling (*CCL3*, *CCL3L3*, *CCL4*, *CCL4L2*) pathways, while the remaining 2 DEGs were lncRNAs (*MEG3* and *RP11-221J22.1*). All 8 shared DEGs had significantly decreased expression in FRAF compared to NORF (Additional file 3) but were significantly increased in FRAM relative to FRAF (Additional file 4).

Interestingly, most of the significant DEGs were exclusively identified in their respective comparison group (Fig. 2A). For example, 14 out of the 23 significant DEGs were unique to the FRAM vs NORM comparison group (Fig. 2C). Only 3 of the 14 unique DEGs (*FCGR3B*, *IL1R2*, and *LRG1*) have been identified in previous frailty studies and an additional 3 DEGs (*PLIN4*, *PLIN5*, and *MGAM*) have been implicated in aging (Additional file 2). Novel DEGs exclusive to the FRAM vs NORM group were associated with many physiological processes such as lipid transport and metabolism (*PLIN4*, *PLIN5*, *MGAM*, and *CTD-3088G3.8*), cell structure (*SAXO2* and *JUP*), and the musculoskeletal system (*SAXO2*, *JUP*, *ALPL*, *PLIN4*, and *PLIN5*) (Additional file 2).

Thirty-six out of the 47 significant DEGs in the FRAF vs NORF comparison group were exclusively identified in this group (Fig. 2D, Additional file 3). Of these 36 exclusively identified DEGs, 11 (*CST7*, *G0S2*, *GPR15*, *GABARAPL1*, *PRF1*, *SMAD7*, *OTUD1*, *S1PR5*, *PDGFD*, *TGFBR3*, and *NR4A3*) have been previously associated with frailty (Additional file 3), while the remaining 29 DEGs were novel. These novel DEGs serve roles in multiple biological functions including but not limited to signal transduction, reactive oxygen species regulation, circadian rhythm, and cellular structure and maintenance (Additional file 3). Additionally, some of the novel DEGs have also been previously described in aging (*LRRN3*, *ZFP36L2*, *ABCA2*, *ID2*, *GABARAPL1*, and *NKG7*, see Additional file 3). Consistent with one of the hallmarks of frailty pathophysiology, many of the novel DEGs exclusively identified in the FRAF vs NORF comparison group were associated with modulating immune and inflammatory responses (*ZFP36L2*, *KLF9*, *BHLHE40*, *MATK*, *TSC22D3*, *ID2*, *NKG7*, *GFI1*, *PTGER4*, *PER1*, *DDIT4*, and *TNFSF9*) and had significantly decreased expression in FRAF compared to NORF (Additional file 3).

Seventy-three significant DEGs were unique to the FRAM vs FRAF comparison group (Fig. 2B, Additional file 4). Only 17 of the 73 exclusive DEGs (*IL1A*, *CXCL3*, *CXCL2*, *CXCL8*, *TNF*, *NFKBIA*, *IL1RN*, *PPP1R15A*, *PTX3*, *ICAM1*, *RIPK2*, *SOD2*, *CYP27A1*, *PPIF*, *SLC2A6*, *FTH1*, and *SULF2*) from the FRAM vs FRAF comparison have been described in frailty literature (Additional file 4). Similarly to the other frailty sex comparison groups, several novel DEGs exclusive to the FRAM vs FRAF group also have putative roles in aging (*ATF3*, *BCL2A1*, *FCAR*, *IER2*, *IER3*, *PDZK1IP1*, and *SSPN*, see Additional file 4). Novel DEGs were also associated with proteostasis, mitochondrial function, metabolism, signal transduction, cell structure, and other biological processes (Additional file 4). Additionally, many of the novel and exclusive DEGs to the FRAM vs FRAF group have multiple roles in frailty and sarcopenia-related pathological processes such as inflammation (*TNFAIP3*, *BCL2A1*, *ZFP36*, *CCL20*, *DUSP1*, *FCAR*, *RNF19B*, *CCRL2*, *CLEC4D*, *FTH1*, and *SIGLEC14*), musculoskeletal system (*ATF3*, *DUSP1*, *TNFAIP3*, *FCAR*, *PPIF*, *MARCKS*, *SSPN*, *KLF10*, and *TANC1*), and cell cycle and apoptosis (*IER3*, *ATF3*, *TNFAIP3*, *DUSP1*, *BCL2A1*, *H1F0*, *PPIF*, *PUDP*, and *ERCC6L*) (Additional file 4). Of note, we also found that 27 of the 61 novel FRAM vs FRAF specific DEGs were noncoding RNAs (Additional file 4), while the FRAM vs NORM and FRAF vs NORF comparison groups only had 1 significant noncoding RNA, respectively (FRAM vs NORM = *FAM157A*; FRAF vs NORF = *AC131056.3*) (Additional files 2 and 3). These results suggest sex-specific gene expression changes associated with frailty occur in midlife.

### Frailty and sex pathway analyses

To discover sex-specific biological and molecular pathways altered in frail, middle-aged individuals, IPA was used to identify significant canonical pathways, while PAGE analysis was used to identify significant GO terms associated with biological processes, molecular functions, and cellular processes. Forty-four IPA canonical pathways were significant in the FRAM vs NORM comparison, 113 in the FRAF vs NORF comparison, 93 in the FRAM vs FRAF comparison, and 88 in the NORM vs NORF comparison (Additional files 6,7,8, and 9). PAGE analyses identified 28 GO biological processes, 20 GO molecular functions, and 9 GO cellular components were significant in the FRAM vs NORM comparison group (Additional files 10,11, and 12). In FRAF vs NORF, 91 GO biological processes, 44 GO molecular functions, and 20 cellular components were significant (Additional files 13,14, and 15). Sixty GO biological processes, 39 GO molecular functions, and 22 GO cellular components were significant in the FRAM vs FRAF comparison group (Additional files 16,17, and 18). Finally, 27 GO biological processes, 21 GO molecular functions, and 15 GO cellular components were significant in the NORM vs NORF comparison group (Additional files 19,20, and 21).

**Fig. 3:**
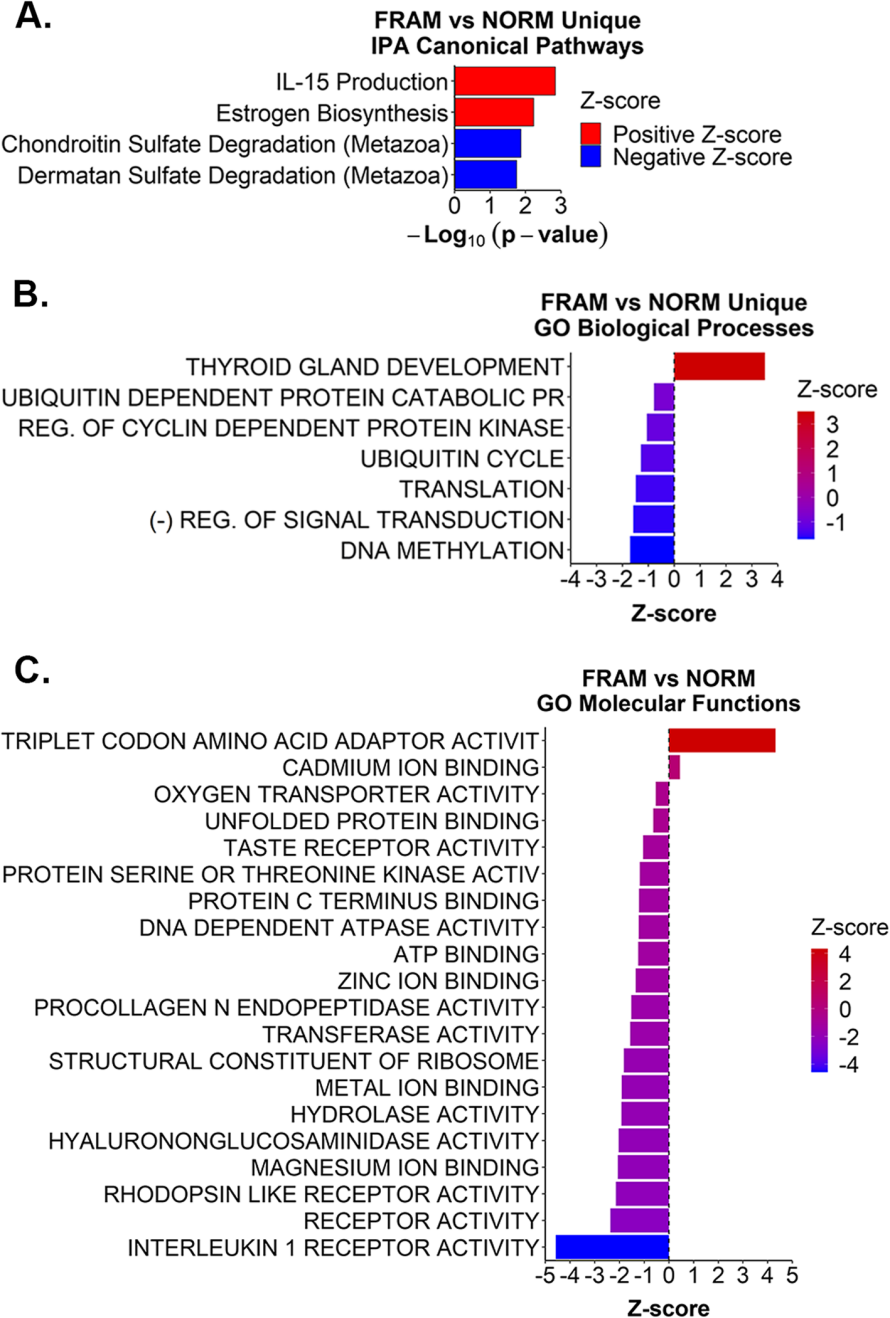
Biological pathways exclusive to FRAM vs NORM. **A**. Significant IPA canonical pathways (y-axis) uniquely identified in the FRAM vs NORM comparison group are plotted by –Log_10_ transformed p-values (x-axis). Red bars indicate a positive Z-score, blue bars indicate a negative Z-score. **B**. Significant GO biological processes (y-axis) exclusively identified in the FRAM vs NORM comparison group, plotted by Z-score (x-axis). **C**. All significant GO molecular functions (y-axis) identified in the FRAM vs NORM group, plotted by Z-score (x-axis). Refer to Additional files 6 and 10, 11, 12 for complete lists of IPA canonical pathways and GO terms. Abbreviations: “(-)” = negative; “REG.” = regulation

**Fig. 4:**
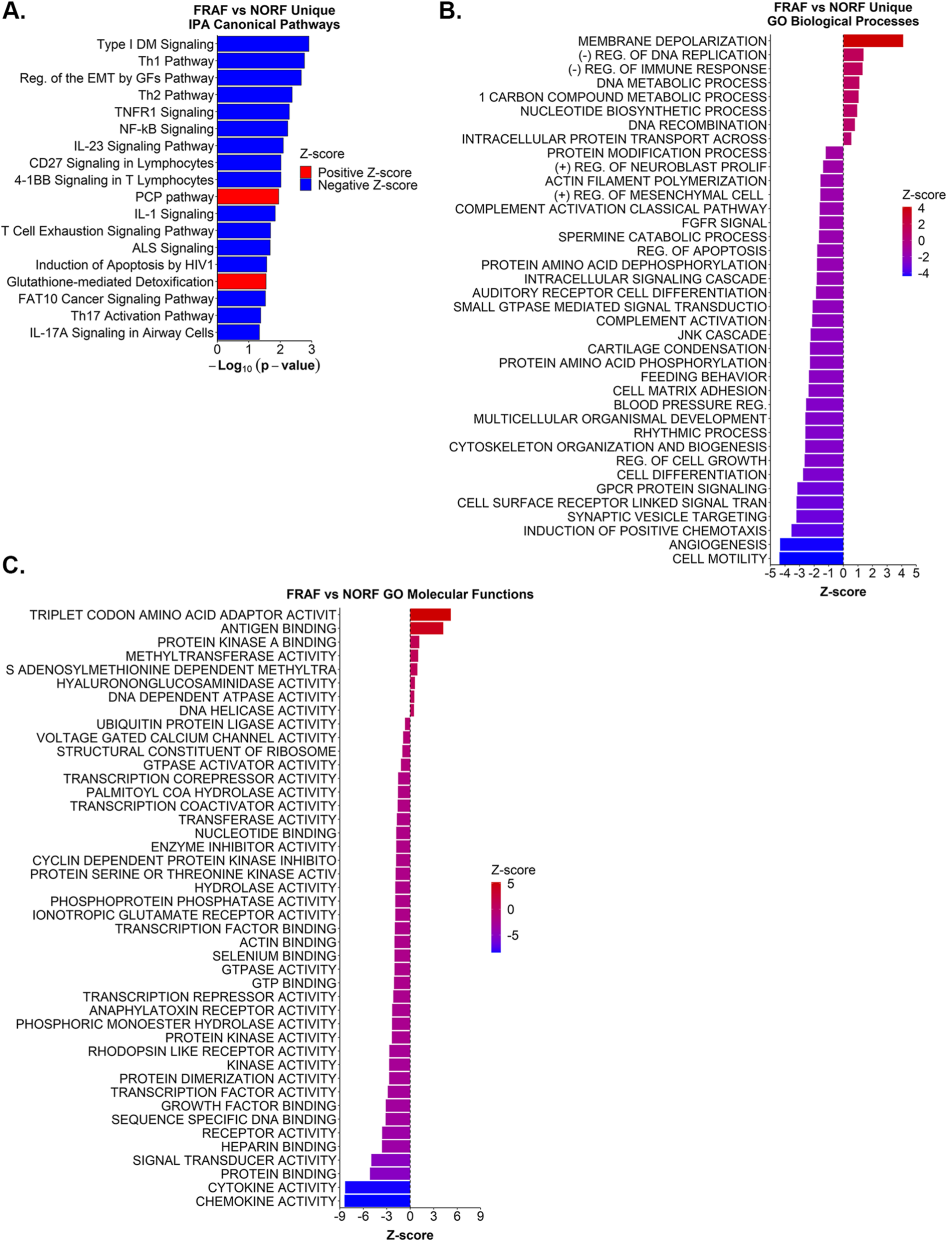
Biological pathways exclusive to FRAF vs NORF. **A**. Significant IPA canonical pathways (y-axis) uniquely identified in the FRAF vs NORF comparison group are plotted by –Log_10_ transformed p-values (x-axis). **B**. Significant GO biological processes (y-axis) exclusively identified in the FRAF vs NORF comparison group, plotted by Z-score (x-axis). **C**. All significant GO molecular functions (y-axis) identified in the FRAF vs NORF group, plotted by Z-score (x-axis). Refer to Additional files 7 and 13, 14, 15 for complete lists of IPA canonical pathways and GO terms. Abbreviations: “Reg. of the EMT by GFs Pathway” = Regulation of the Epithelial Mesenchymal Transition by Growth Factors Pathway; “DM” = Diabetes Mellitus; “ALS” = Amyotrophic lateral sclerosis; “(-)” = negative; “( +)” = positive; “REG.” = regulation; “FGFR” = fibroblast growth factor receptor; “GPCR” = G-protein coupled receptor

**Fig. 5:**
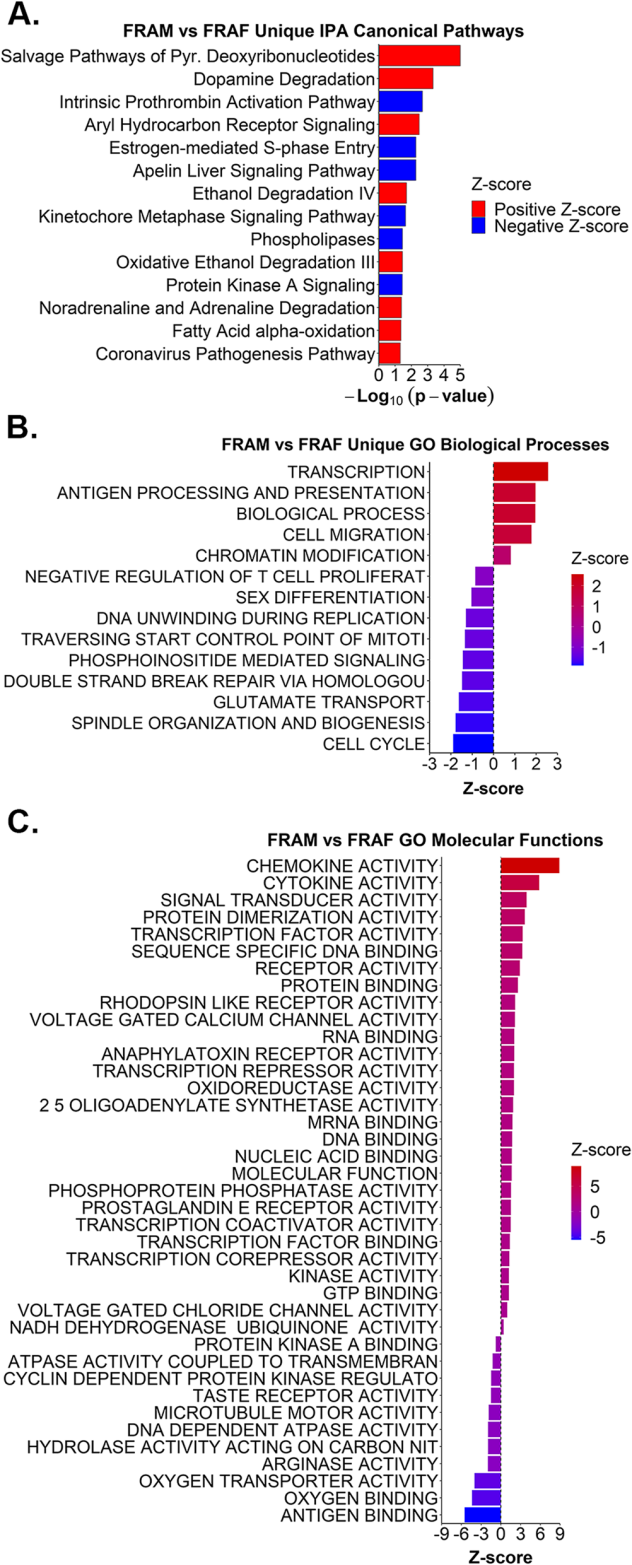
Biological pathways exclusive to FRAM vs FRAF. **A**. Significant IPA canonical pathways (y-axis) uniquely identified in the FRAM vs FRAF comparison group are plotted by –Log_10_ transformed p-values (x-axis). **B**. Significant GO biological processes (y-axis) exclusively identified in the FRAM vs FRAF comparison group, plotted by Z-score (x-axis). **C**. All significant GO molecular functions (y-axis) identified in the FRAM vs FRAF group, plotted by Z-score (x-axis). Refer to Additional files 8 and 16, 17, 18 for complete lists of IPA canonical pathways and GO terms. Abbreviations: “Pyr.” = pyrimidine

We evaluated the IPA canonical pathways and GO biological processes for overlaps among the 4 frailty comparison groups (Additional file 22). Nineteen canonical pathways were shared among all 4 frailty comparison groups (Additional file 22A), while only 1 GO biological process was shared between all 4 (Additional file 22B). Five canonical pathways and biological processes, respectively, were shared between the FRAM vs NORM, FRAF vs NORF, and FRAM vs FRAF groups (Additional file 22). We also assessed exclusive pathways and GO biological processes per respective comparison group to identify sex-specific pathway differences by frailty status. When examining IPA canonical pathways, 12 were exclusively identified in FRAM vs NORM, 30 in FRAF vs NORF, and 17 in FRAM vs FRAF (Additional file 22A, Additional files 6,7, and 8). To identify pathways predicted to be activated or inhibited, we focused our attention to exclusive IPA canonical pathways with a calculated Z-score. Based on this criteria, 4 canonical pathways were only identified in FRAM vs NORM (Fig. 3A, Additional file 6), 18 were unique to FRAF vs NORF (Fig. 4A, Additional file 7), and 14 were exclusive to FRAM vs FRAF (Fig. 5A, Additional file 8). For the GO biological processes, 7 were unique to FRAM vs NORM (Fig. 3B, Additional file 22B, Additional file 10), 38 in FRAF vs NORF (Fig. 4B, Additional file 22B, Additional file 13), and 14 in FRAM vs FRAF (Fig. 5B, Additional file 22B, Additional file 16).

### Canonical pathways and biological processes in frail males

To get a better understanding of the pathophysiology of frailty in males, we examined the IPA canonical pathways exclusively identified in the FRAM vs NORM group. These pathways were associated with glycosaminoglycan (GAG) catabolism (“Chondroitin Sulfate Degradation” and “Dermatan Sulfate Degradation”), estrogen (“Estrogen Biosynthesis), and inflammation (“IL-15 Production”) (Fig. 3A). Deficiencies in chondroitin sulfate (CS) and dermatan sulfate (DS) degradation enzymes result in the accumulation of CS, DS, and other GAGs in the lysosome [22]. This lysosomal GAG accumulation can initiate several secondary molecular cascades that disrupt signaling pathways regulating inflammation and importantly skeletal structure and integrity [22]. Furthermore, male estrogen levels have a protective skeletal effect and mediates bone biosynthesis through the CS subtype, CS-E [23]. Here reduced CS and DS degradation, and consequent CS/DS accumulation, in FRAM could disrupt proper signaling for skeletal and connective tissue integrity perhaps leading to aberrant inflammation.

Consistent with this idea, we observed GO biological processes uniquely identified in the FRAM vs NORM group were associated with reduced degradative and homeostatic processes as well as reduced signal transduction (Fig. 3B). GO biological processes associated with ubiquitin and proteostasis were down-regulated in FRAM compared to NORM (Fig. 3B). Signaling-related GO biological processes and molecular functions were also reduced in FRAM (Fig. 3B and C). With respect to CS and DS degradation pathways, we also observed the GO molecular functions “Procollagen N endopeptidase activity”, “Hydrolase activity”, and “Hyalurononglucosaminidase activity” were down-regulated in FRAM (Fig. 3C). These findings suggest unique biological processes and molecular functions are altered with frailty in males.

### Canonical pathways and biological processes exclusive to frail women

IPA canonical pathways that were only identified in the FRAF vs NORF group were largely associated with immune responses and inflammation (Fig. 4A). For example, we identified T helper cell pathways, interleukin signaling pathways, and lymphocyte signaling pathways were significantly altered in FRAF compared to NORF (Fig. 4A). Interestingly, all these pathways are down-regulated in FRAF. GO biological processes uniquely identified in the FRAF vs NORF group were consistent with these observations (Fig. 4B). The GO biological processes “Negative regulation of immune response” was up-regulated, while “Complement activation classical pathway” and “Complement activation” were down-regulated (Fig. 4B). GO molecular functions revealed “Antigen binding” was up-regulated in FRAF compared to NORF, while “Cytokine activity” and “Chemokine activity” were down-regulated (Fig. 4C).

Additional pathways were associated with signal transduction and cellular structure (Fig. 4A). For example, the “TNFR1 signaling” and “NF-kB signaling” pathways have crucial roles in both inflammation and signal transduction. Other signal transduction pathways and biological processes were also associated with frailty in females and in general were reduced with frailty. Consistent with signal transduction roles, GO biological processes uniquely identified in the FRAF vs NORF group included “Small GTPase mediated signal transduction” and “G protein coupled receptor protein signaling” (Fig. 4B). Apart from the “Intracellular protein transport across a membrane” GO term, all other GO biological processes were significantly reduced (Fig. 4B). GO molecular functions associated with signal transduction included reduced “GTPase activator activity”, “GTP binding”, and “Signal transducer activity” (Fig. 4C). There were several GO biological processes and molecular functions that were associated with regulation of the actin cytoskeleton and cartilage, all of which were significantly down-regulated in FRAF (Fig. 4B and C).

### Canonical pathways and GO biological processes exclusive to frail men vs frail women

Many of the pathways exclusively identified in the FRAM vs FRAF group were predominantly associated with cell cycle and metabolic processes (Fig. 5), including the “Aryl hydrocarbon receptor signaling” pathway (Fig. 5A), “Kinetochore metaphase signaling pathway”, and “Estrogen-mediated S-phase entry” (Fig. 5A). GO biological processes exclusively identified in the FRAM vs FRAF comparison group included up-regulation of cell cycle, chromatin remodeling, and DNA repair (Fig. 5B). We also observed the GO molecular functions “Cyclin dependent protein kinase regulator activity” and “microtubule motor activity” were down-regulated in FRAM vs FRAF (Fig. 5C). The metabolic-related pathways “Dopamine degradation”, “Noradrenaline and adrenaline degradation”, and “Fatty acid alpha-oxidation” all had positive Z-scores (Fig. 5A), suggesting that catabolism could be up-regulated in FRAM compared to FRAF. Related GO molecular functions such as “Oxidoreductase activity” and “NADH dehydrogenase ubiquinone activity” were also up-regulated (Fig. 5C).

The “Salvage pathways of pyrimidine deoxyribonucleotides” had a positive Z-score (Fig. 5A). Pathogens and viruses can utilize pyrimidine deoxyribonucleotide salvage pathways to self-replicate [24]. Interestingly, we observed that the “Coronavirus pathogenesis pathway” was (Fig. 5A) up-regulated in FRAM compared to FRAF. Consistent with this finding, the other immune response GO biological processes “Antigen processing and presentation” and “Negative regulation of T cell proliferation” and molecular functions associated with inflammation and immune response were exclusive to the FRAM vs FRAF comparison group (Fig. 5B-C). These data show important differences between frail males and females in pathways related to coronavirus pathogenesis, immune responses, and inflammation.

## Discussion

This study examined sex differences in global gene expression changes associated with frailty in a middle-aged cohort. We found sex specific novel genes and biological pathways implicated in frailty pathophysiology. Compared to non-frail women, genes and pathways associated with inflammation were down-regulated in frail women, while frail men had molecular changes related to proteostasis and musculoskeletal structure and integrity compared to non-frail men. Transcriptome differences between frail men and frail women reflected processes associated with cell cycle regulation, metabolism, and immune responses.

We aimed to identify molecular targets and pathways that could contribute to previously observed sex-specific health disparities in frailty. While some of the uniquely identified significant genes from each respective comparison group have been previously described in frailty literature, many of the genes appear to be novel and may serve important roles in frailty-associated pathological mechanisms. For example, *JUP* and *ALPL* were exclusively identified in the FRAM vs NORM comparison and have been associated with musculoskeletal development [25] and bone mineralization [26], respectively. *TSC22D3*, a gene uniquely identified in the FRAF vs NORF comparison, codes for the glucocorticoid-induced leucine zipper (GILZ) protein, in which decreased expression has been associated with inflammaging in mice [27]. *IER3*, uniquely identified in the FRAM vs FRAF comparison group, was previously associated with mortality [28] and can inhibit NF-κB signaling in response to TNF-α activation via negative feedback loop [29]. Therefore, several of the frailty-associated genes identified in our analysis may have physiological relevance in frailty.

Notably, we also observed over a third of the novel genes exclusively found in the FRAM vs FRAF comparison were noncoding RNAs (ncRNAs). Limited studies have examined noncoding RNAs in frailty and have mostly focused on miRNA expression, many of which are involved in inflammatory processes [30, 31]. Consistent with these findings, some of the novel ncRNAs exclusively identified in the FRAM vs FRAF comparison group have been previously associated with inflammatory processes. For example, *LINC00936* and *LINC00528* were recently predicted to interact with *TLR2* and the Toll-like receptor signaling pathway in acute myocardial infarction [32]. Another study showed overexpression of *LINC00936* in cardiomyocytes resulted in significantly reduced amounts of IL-10 and higher amounts of IL-6, IL-1β, and TNFα [33]. Collectively, our results suggest ncRNAs could potentially contribute to sex-specific differences associated with chronic inflammation in aging and frailty. Given that a miRNA panel has been recommended to be incorporated into a core biomarker panel for frailty [34], future studies will be needed to understand the contributions of miRNAs and other ncRNAs driving sex-specific differences in frailty.

Pathways unique to the FRAM vs NORM comparison group suggest processes regulating musculoskeletal and connective tissue physiology could be reduced in frail men. Increased risk of fractures and bone deficits as well as reduced muscle mass and strength have been well-documented in frailty for men and women (reviewed in [35]). Another study examined sex-specific sarcopenia prevalence in individuals after hip fracture and found sarcopenia was significantly higher in men compared to women [36]. Hormonal imbalance has been proposed to contribute to abnormal musculoskeletal physiology in frailty [35]. The estrogen biosynthesis pathway was uniquely identified in the FRAM vs NORM comparison group and was upregulated in FRAM compared to NORM. Studies examining the relationship between estrogen, musculoskeletal physiology, and frailty in men have produced conflicting results. Notably, a recent study found frail men with greater baseline estradiol concentrations had a decreased likelihood of improving their frailty status [37]. Whether increased estrogen biosynthesis confers a protective or detrimental effect in middle-aged frail men is unclear and requires additional investigation.

Another potential mechanism contributing to musculoskeletal deficits includes chronic low-grade inflammation [35]. For example, IL-15 has been implicated in bone-muscle cross-talk [35]. The IL-15 production pathway was up-regulated in FRAM compared to NORM. Interestingly, the accumulation of GAGs such as DS has been shown to activate inflammatory processes [38], and a recent proteomic study in frailty revealed glycosaminoglycan metabolism was one of the top pathways associated with frailty [39]. In line with these observations, we observed down-regulation of CS and DS degradation pathways in FRAM. CS and DS degradation mechanisms have been unexplored in frailty. Given the broad downstream molecular and cellular effects of GAG accumulation, it is possible that aberrant signal transduction observed in frailty could be due to GAGs.

Pathways uniquely identified in the FRAF vs NORF comparison group suggest reduced inflammation could be mediated through reduced T cell and interleukin signaling. BHLHE40 is a transcription factor with roles in Th1 and Th17 effector and pathogenic functions, as well as supporting mitochondrial fitness and metabolism in CD8 + tissue-resident memory cells and tumor-infiltrating lymphocytes [40]. In line with these observations, we observed down-regulation of multiple T helper cell pathways including Th1 and Th17 activation in FRAF compared to NORF. *TNFSF9* codes for 4-1BB or CD137 ligand, which has been implicated in the activation, response, maintenance, and survival of various immune cells, especially for T cells (reviewed in [41]). Consistent with these roles, we also observed down-regulation of the 4-1BB signaling in T lymphocytes pathway in FRAF relative to NORF.

This seems to conflict with current knowledge of inflammation in frailty, where pro-inflammatory cytokines and processes are upregulated while anti-inflammatory processes are reduced (reviewed in [42, 43]). However, our results still support the notion that aberrant immunological and inflammatory processes are more pronounced in women compared to men [44]. Future work lies in determining how these inflammatory processes are dysregulated in middle-aged frail women.

Pathways exclusive to the FRAM vs FRAF comparison group suggested increased catabolism and down-regulation of cell cycle pathways in FRAM relative to FRAF. Reduced energy metabolism has been linked to frailty [45]. We observed pathways associated with metabolic degradation were upregulated in FRAM relative to FRAF. For example, the dopamine degradation pathway was upregulated in FRAM vs FRAF. One of the intermediate products of the dopamine degradation pathway is 3,4-dihydroxyphenylacetic acid. A previous study showed dihydroxyphenyl acetic acid was decreased in pre-frail women but not men [45]. How changes in these metabolic degradation pathways contribute to pathophysiology differences between frail men and frail women will require additional investigation.

Cell cycle control is one of the hallmarks of aging. A previous study found increased frailty, low BMI, and 9 upregulated transcripts with roles in cell cycle, inflammation, and mitochondrial function were the best predictors of mortality [28]. In the present study, we found cell cycle related pathways were down-regulated in FRAM relative to FRAF, suggesting that cell cycle progression could be inhibited in FRAM. Cell cycle arrest is one of the key features of cellular senescence, which has been considered one of the mechanisms contributing to chronic inflammation in frailty [46]. Senescent cells can develop a senescence-associated secretory phenotype (SASP), which can secrete various inflammatory molecules such as IL-1α, IL-1β, IL-6, IL-8, and TNF-α [34]. We observed significantly increased gene expression for *IL1A* (IL-1α), *CXCL8* (IL-8), *TNF* (TNF-α), and other pro-inflammatory genes in FRAM relative to FRAF.

Thus, it is tempting to speculate that there may be differences in immunosenescence between middle-aged frail men and frail women. A previous review proposed that men could undergo greater and more accelerated immunosenescence compared to women, potentially contributing to aging and survival differences [11]. Consistent with this idea, men have faster extrinsic epigenetic age acceleration, which is an epigenetic aging measure that captures immunosenescence [47]. Functional validation studies will be needed to further investigate these differences.

On a timely note, we observed the Coronavirus pathogenesis pathway was predicted to be upregulated in FRAM compared to FRAF and was uniquely identified in this respective comparison group. A recent review reported frailty was linked to coronavirus disease 2019 (COVID-19) severity risk and mortality [48]. Importantly, sex differences in COVID-19 severity and mortality have also been documented, where men have greater severe COVID-19 prevalence and mortality [49]. Many of the upregulated genes annotated in the Coronavirus pathogenesis pathway, such as *IL6* and *CCL2*, are associated with hypercytokinemia or cytokine storm, a systemic hyper-inflammatory state that has been shown to influence COVID-19 disease severity [49]. Related to our findings, women have a reduced chance to progress into systemic hyper-inflammatory states including cytokine storms [49]. Additionally, a study examining COVID-19 patients with moderate disease found that men had increased levels of innate immune cytokines and robust induction of non-classical monocytes, while women had robust T cell activation [49]. It is plausible that increased susceptibility to viral infections and cytokine storm in frail men could begin to manifest in midlife. This earlier manifestation could potentially contribute to early mortality in frail men.

While our study provides novel insight into sex-specific transcriptome changes in middle-aged frailty, there are some limitations. In this study, the FRAIL scale was used to classify frailty [13]. There are multiple methods available to assess frailty such as the frailty phenotype and the frailty index [48], which are more commonly utilized. However, these indexes require hospital settings, which are not applicable for community-based research and clinics. We previously demonstrated validity of the FRAIL scale in the HANDLS cohort [7] and this frailty measure has been utilized extensively and successfully in community-based cohorts and patients [13]. Current transcriptome studies in frailty have only examined European and Asian older adult (≥ 65 years old) cohorts [30, 50–57]. No transcriptome studies have examined frailty in diverse cohorts or frailty in midlife. Here, our results shed new light on the potential molecular drivers of sex-based differences in frailty in a diverse cohort. Therefore, although our sample size is small the results from this study are still novel based on our methodology, diverse sample demographics, and age of our cohort.

## Conclusions

Our transcriptome-wide results revealed sex-specific differences associated with frailty in midlife. This study builds on previous frailty work by confirming musculoskeletal, metabolic, and immunological and inflammatory processes are also disrupted in middle-aged frail individuals. Importantly, our work provides novel insight on candidate genes and biological pathways that could contribute to molecular differences in inflammatory, musculoskeletal, and other frailty pathophysiological profiles between middle-aged men and women. This work highlights the importance of examining frailty in middle-aged cohorts, before the older ages traditionally evaluated for frailty. By evaluating gene expression changes in a middle-aged frail cohort, we can begin to advance knowledge on frailty progression and identify potential therapeutic targets to prevent frailty.

## Supplementary Information

**Additional file 1.** Bioinformatics analysis pipeline overview. Refer to the methods section for more detailed information.

**Additional file 2.** Significant, differentially expressed genes in FRAM vs NORM. Ensembl ID, gene symbol, Log_2_fold change, padj value, gene type, and associated GO biological processes are listed for each respective DEG. Gene type and GO biological processes were retrieved from Ensembl’s BioMart (Ensembl Genes 104, Ensembl human genome version GRCh38.p13) as of August 10^th^, 2021. Information on whether genes were uniquely identified in the FRAM vs NORM comparison group is provided in the column “Unique to FRAM vs NORM”. For genes designated as “YES” in the “Unique to FRAM vs NORM” column, information on whether they were previously identified in aging and/or frailty studies are provided in the column “Previously identified in aging and/or frailty studies”. References for previously identified genes are listed in the “References” column. “N/A” = information not available.

**Additional file 3.** Significant, differentially expressed genes in FRAF vs NORF. Ensembl ID, gene symbol, Log_2_ fold change, padj value, gene type, and associated GO biological processes are listed for each respective DEG. Gene type and GO biological processes were retrieved from Ensembl’s BioMart (Ensembl Genes 104, Ensembl human genome version GRCh38.p13) as of August 10^th^, 2021. Information on whether genes were uniquely identified in the FRAF vs NORF comparison group is provided in the column “Unique to FRAF vs NORF”. For genes designated as “YES” in the “Unique to FRAF vs NORF” column, information on whether they were previously identified in aging and/or frailty studies are provided in the column “Previously identified in aging and/or frailty studies”.  References for previously identified genes are listed in the “References” column. “N/A” = information not available.

**Additional file 4.** Significant, differentially expressed genes in FRAM vs FRAF. Ensembl ID, gene symbol, Log_2_ fold change, padj value, gene type, and associated GO biological processes are listed for each respective DEG. Gene type and GO biological processes were retrieved from Ensembl’s BioMart (Ensembl Genes 104, Ensembl human genome version GRCh38.p13) as of August 10^th^, 2021. Information on whether genes were uniquely identified in the FRAM vs FRAF comparison group is provided in the column “Unique to FRAM vs FRAF”. For genes designated as “YES” in the “Unique to FRAM vs FRAF” column, information on whether they were previously identified in aging and/or frailty studies are provided in the column “Previously identified in aging and/or frailty studies”.  References for previously identified genes are listed in the “References” column. “N/A” = information not available.

**Additional file 5.** Significant, differentially expressed genes in NORM vs NORF. Ensembl ID, gene symbol, Log_2 _fold change, padj value, gene type, and associated GO biological processes are listed for each respective DEG. Gene type and GO biological processes were retrieved from Ensembl’s BioMart (Ensembl Genes 104, Ensembl human genome version GRCh38.p13) as of August 10^th^, 2021. Information on whether genes were uniquely identified in the NORM vs NORF comparison group is provided in the column “Unique to NORM vs NORF”. “N/A” = information not available.

**Additional file 6.** Significant IPA Canonical Pathways in FRAM vs NORM. For each significant pathway, the -Log_10 _(p-value), Z-score, and gene associated with the respective pathway (column “Molecules”) are provided. Pathways that were uniquely identified in the FRAM vs NORM comparison group are denoted in the “Unique to FRAM vs NORM” column.“N/A” = information not available.

**Additional file 7.** Significant IPA Canonical Pathways in FRAF vs NORF. For each significant pathway, the -Log_10 _(p-value), Z-score, and gene associated with the respective pathway (column “Molecules”) are provided. Pathways that were uniquely identified in the FRAF vs NORF comparison group are denoted in the “Unique to FRAF vs NORF” column. “N/A” = information not available.

**Additional file 8.** Significant IPA Canonical Pathways in FRAM vs FRAF. For each significant pathway, the -Log_10_ (p-value), Z-score, and gene associated with the respective pathway (column “Molecules”) are provided. Pathways that were uniquely identified in the FRAM vs FRAF comparison group are denoted in the “Unique to FRAM vs FRAF” column. “N/A” = information not available.

**Additional file 9.** Significant IPA Canonical Pathways in NORM vs NORF. For each significant pathway, the -Log_10 _(p-value), Z-score, and gene associated with the respective pathway (column “Molecules”) are provided. Pathways that were uniquely identified in the NORM vs NORF comparison group are denoted in the “Unique to NORM vs NORF” column. “N/A” = information not available.

**Additional file 10.** Significant gene ontology (GO) biological processes in FRAM vs NORM. The complete GO annotation description, GO accession number, and Z-score are provided for all significant GO biological process terms identified in the FRAM vs NORM comparison group. GO biological process terms uniquely identified in the FRAM vs NORM comparison group are denoted in the “Unique to FRAM vs NORM” column.

**Additional file 11.** Significant gene ontology (GO) molecular functions in FRAM vs NORM. The complete GO annotation description, GO accession number, and Z-score are provided for all significant GO molecular function terms identified in the FRAM vs NORM comparison group. GO molecular function terms uniquely identified in the FRAM vs NORM comparison group are denoted in the “Unique to FRAM vs NORM” column.

**Additional file 12.** Significant gene ontology (GO) cellular components in FRAM vs NORM. The complete GO annotation description, GO accession number, and Z-score are provided for all significant GO cellular component terms identified in the FRAM vs NORM comparison group. GO cellular component terms uniquely identified in the FRAM vs NORM comparison group are denoted in the “Unique to FRAM vs NORM” column.

**Additional file 13.** Significant gene ontology (GO) biological processes in FRAF vs NORF. The complete GO annotation description, GO accession number, and Z-score are provided for all significant GO biological process terms identified in the FRAF vs NORF comparison group. GO biological process terms uniquely identified in the FRAF vs NORF comparison group are denoted in the “Unique to FRAF vs NORF” column.

**Additional file 14.** Significant gene ontology (GO) molecular functions in FRAF vs NORF. The complete GO annotation description, GO accession number, and Z-score are provided for all significant GO molecular function terms identified in the FRAF vs NORF comparison group. GO molecular function terms uniquely identified in the FRAF vs NORF comparison group are denoted in the “Unique to FRAF vs NORF” column.

**Additional file 15.** Significant gene ontology (GO) cellular components in FRAF vs NORF. The complete GO annotation description, GO accession number, and Z-score are provided for all significant GO cellular component terms identified in the FRAF vs NORF comparison group. GO cellular component terms uniquely identified in the FRAF vs NORF comparison group are denoted in the “Unique to FRAF vs NORF” column.

**Additional file 16.** Significant gene ontology (GO) biological processes in FRAM vs FRAF. The complete GO annotation description, GO accession number, and Z-score are provided for all significant GO biological process terms identified in the FRAM vs FRAF comparison group. GO biological process terms uniquely identified in the FRAM vs FRAF comparison group are denoted in the “Unique to FRAM vs FRAF” column.

**Additional file 17.** Significant gene ontology (GO) molecular functions in FRAM vs FRAF. The complete GO annotation description, GO accession number, and Z-score are provided for all significant GO molecular function terms identified in the FRAM vs FRAF comparison group. GO molecular function terms uniquely identified in the FRAM vs FRAF comparison group are denoted in the “Unique to FRAM vs FRAF” column.

**Additional file 18.** Significant gene ontology (GO) cellular components in FRAM vs FRAF. The complete GO annotation description, GO accession number, and Z-score are provided for all significant GO cellular components terms identified in the FRAM vs FRAF comparison group. GO cellular components terms uniquely identified in the FRAM vs FRAF comparison group are denoted in the “Unique to FRAM vs FRAF” column.

**Additional file 19.** Significant gene ontology (GO) biological processes in NORM vs NORF. The complete GO annotation description, GO accession number, and Z-score are provided for all significant GO biological process terms identified in the NORM vs NORFcomparison group. GO biological process terms uniquely identified in the NORM vs NORF comparison group are denoted in the “Unique to NORM vs NORF” column.

**Additional file 20.** Significant gene ontology (GO) molecular functions in NORM vs NORF. The complete GO annotation description, GO accession number, and Z-score are provided for all significant GO molecular function terms identified in the NORM vs NORF comparison group. GO molecular function terms uniquely identified in the NORM vs NORF comparison group are denoted in the “Unique to NORM vs NORF” column.

**Additional file 21.** Significant gene ontology (GO) cellular components in NORM vs NORF. The complete GO annotation description, GO accession number, and Z-score are provided for all significant GO cellular components terms identified in the NORM vs NORF comparison group. GO cellular components terms uniquely identified in the NORM vs NORF comparison group are denoted in the “Unique to NORM vs NORF” column.

**Additional file 22.** Significant overlapping and exclusive biological pathways. Venn diagrams comparing the lists of A. significant IPA canonical pathways and B. significant GO biological processes identified from each respective comparison group. Pathways and GO terms overlapping between all 4 groups are listed to the right of each respective venn diagram. FRAM vs NORM = yellow, FRAF vs NORF = blue, FRAM vs FRAF = purple, and NORM vs NORF = gray (as a reference group).

## Data Availability

The raw fastq data is available at NCBI GEO repository (accession number GSE129534, https://www.ncbi.nlm.nih.gov/geo/query/acc.cgi?acc=GSE129534). The master counts tables and scripts are available upon request. All other data generated or analyzed during the study are included in this published article and respective supplementary information files.

## Abbreviations

HANDLS: Healthy Aging in Neighborhoods of Diversity across the Life Span
FRAF: Frail females
NORF: Non-frail females
FRAM: Frail males
NORM: Non-frail males
AA: African American
FRAIL: Fatigue, resistance, ambulation, illnesses, and loss of weight
FDR: False discovery rate
PAGE: Parametric Analysis of Gene Set Enrichment
IPA: Ingenuity Pathway Analysis
GO: Gene ontology
DEGs: Differentially expressed genes
lncRNA: Long noncoding RNA
GAG: Glycosaminoglycan
CS: Chondroitin sulfate
DS: Dermatan sulfate
GILZ: Glucocorticoid-induced leucine zipper
ncRNAs: Noncoding RNAs
miRNA: MicroRNA
NK: Natural killer
Th: T helper
SASP: Senescence-associated secretory phenotype
COVID-19: Coronavirus disease 2019
padj: P-adjusted value
(-): Negative
REG: Regulation
Reg. of the EMT by GFs Pathway: Regulation of the Epithelial Mesenchymal Transition by Growth Factors Pathway
DM: Diabetes Mellitus
ALS: Amyotrophic lateral sclerosis
(+): Positive
FGFR: Fibroblast growth factor receptor
GPCR: G-protein coupled receptor
Pyr: Pyrimidine
