# Supplementary figures and images for: Sex-specific transcriptome differences in a middle-aged frailty cohort
Source: BMC Geriatr. 2022 Aug 9;22:651. doi: 10.1186/s12877-022-03326-7 (PMC9361278; doi:10.1186/s12877-022-03326-7)

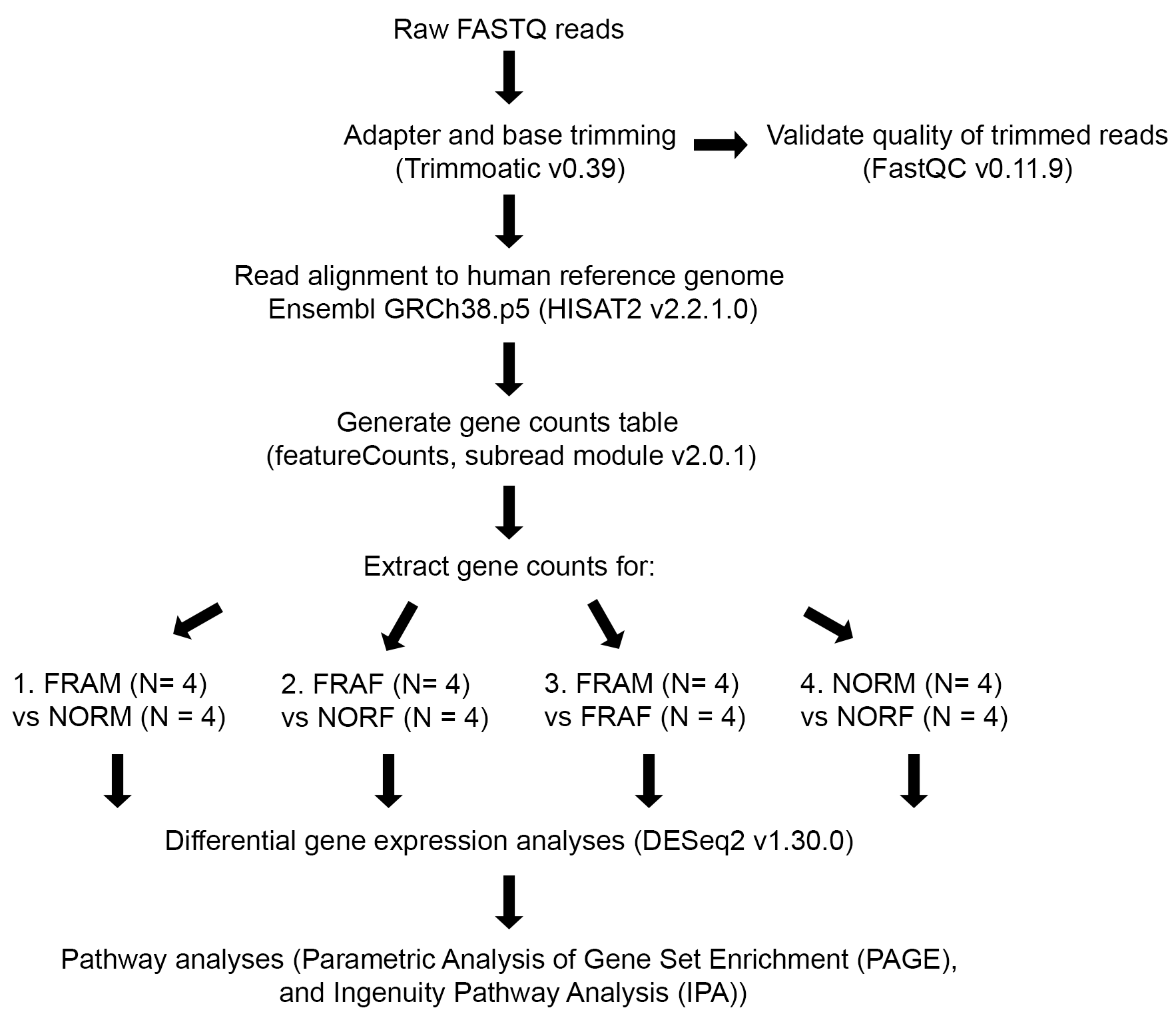

Supplement: Supplementary file 1 — Additional file 1. Bioinformatics analysis pipeline overview. Refer to the methods section for more detailed information. [file 12877_2022_3326_MOESM1_ESM.tif]

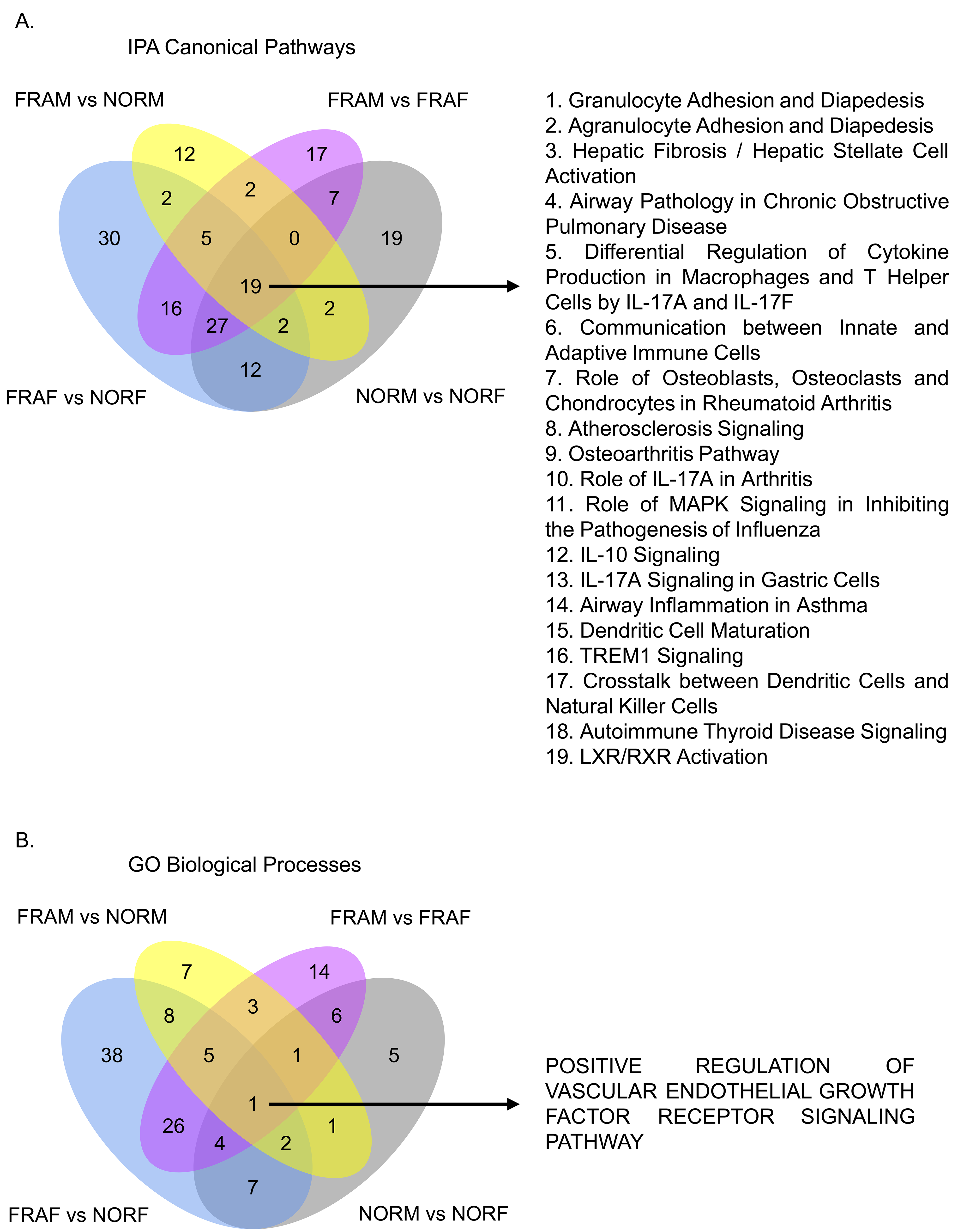

Supplement: Supplementary file 22 — Additional file 22. Significant overlapping and exclusive biological pathways. Venn diagrams comparing the lists of A. significant IPA canonical pathways and B. significant GO biological processes identified from each respective comparison group. Pathways and GO terms overlapping between all 4 groups are listed to the right of each respective venn diagram. FRAM vs NORM = yellow, FRAF vs NORF = blue, FRAM vs FRAF = purple, and NORM vs NORF = gray (as a reference group). [file 12877_2022_3326_MOESM22_ESM.tif]
